# Thermal Stress Triggers Broad *Pocillopora damicornis* Transcriptomic Remodeling, while *Vibrio coralliilyticus* Infection Induces a More Targeted Immuno-Suppression Response

**DOI:** 10.1371/journal.pone.0107672

**Published:** 2014-09-26

**Authors:** Jeremie Vidal-Dupiol, Nolwenn M. Dheilly, Rodolfo Rondon, Christoph Grunau, Céline Cosseau, Kristina M. Smith, Michael Freitag, Mehdi Adjeroud, Guillaume Mitta

**Affiliations:** 1 CNRS, Ecologie et Evolution des Interactions, UMR 5244, Perpignan, France; 2 Univ. Perpignan Via Domitia, Ecologie et Evolution des Interactions, UMR 5244, Perpignan, France; 3 Reponse Immunitaire des Macroorganismes et Environnement, Ecologie des Systèmes Marins côtiers, UMR 5119 CNRS-Ifremer-UM2, Montpellier, France; 4 Department of Biochemistry and Biophysics, Center for Genome Research and Biocomputing, Oregon State University, Corvallis, Oregon, United States of America; 5 Institut de Recherche pour le Développement, Unité 227 CoRéUs2 “Biocomplexité des écosystèmes coralliens de l’Indo-Pacifique”, Laboratoire d’excellence CORAIL, Banyuls-sur-Mer, France; Fish Vet Group, Thailand

## Abstract

Global change and its associated temperature increase has directly or indirectly changed the distributions of hosts and pathogens, and has affected host immunity, pathogen virulence and growth rates. This has resulted in increased disease in natural plant and animal populations worldwide, including scleractinian corals. While the effects of temperature increase on immunity and pathogen virulence have been clearly identified, their interaction, synergy and relative weight during pathogenesis remain poorly documented. We investigated these phenomena in the interaction between the coral *Pocillopora damicorni*s and the bacterium *Vibrio coralliilyticus*, for which the infection process is temperature-dependent. We developed an experimental model that enabled unraveling the effects of thermal stress, and virulence *vs.* non-virulence of the bacterium. The physiological impacts of various treatments were quantified at the transcriptome level using a combination of RNA sequencing and targeted approaches. The results showed that thermal stress triggered a general weakening of the coral, making it more prone to infection, non-virulent bacterium induced an ‘efficient’ immune response, whereas virulent bacterium caused immuno-suppression in its host.

## Introduction

Greenhouse gas emissions have increased since the industrial revolution, and the exceptionally high concentrations now reached have caused global climate change [1]. One of the major consequences of global climate change is that the overall seawater temperature has increased by a mean value of 0.6°C over the last 100 years [2]. The direct consequences of these changes are mass mortality of species having limited ability to adapt to temperature increase [2], [3], but indirect consequences can include mortalities triggered by temperature-dependent epizootics [4].

Marine disease risk is clearly enhanced under global warming conditions [5], [6], but the causes of the increase in the frequency and severity of epizootics are unclear and probably multi-factorial. It is known that global warming can change host and pathogen repartition to enhance the probability of host/pathogen encounters, or to facilitate new interactions. If some of these new host/pathogen interactions are compatible (susceptible host and/or virulent pathogen), a rapid spread of disease can occur [4]–[7], leading to massive terrestrial and marine vertebrate or invertebrate host die-offs [8]–[13]. In addition, an increase in temperature can diminish host immune abilities, leading to increased susceptibility to disease [14], [15] or increased pathogen virulence [16]–[18]. Taken together, these data show that the emergence of epizootics under climate change conditions is probably a result of multiple factors, the relative contribution and synergy of which must be studied to better understand and predict the epizootic risk in the current context of climate change [5], [19].

Among marine host/pathogen models, the interaction between the coral *Pocillopora damicornis* and the bacterium *Vibrio coralliilyticus* is useful for studying and unraveling the effects of temperature and bacterial virulence in pathogenesis. *P. damicornis* is widely distributed in the Indo-Pacific region [20], [21] and is highly susceptible to a wide range of disturbances [22]–[25], including disease [26]–[29]. *V. coralliilyticus* is a common pathogen [30] whose virulence is temperature-dependent [26]. The genomes of two *V. coralliilyticus* strains were recently sequenced, and a proteomic study performed under a range of temperature conditions has revealed the thermo-dependent expression of several putative virulence factors [31], [32], but their effects on coral physiology remain to be characterized. Recent data obtained using this infection model showed that the bacterium modulates the expression of several immune genes in *P. damicornis* [33], and that in its virulent state the *Vibrio* strongly decreases the expression of a gene encoding the antimicrobial peptide damicornin [34]. In this interaction the symptoms differ depending on the populations or strains involved. When *V. coralliilyticus* YB1 (isolated from Zanzibar) contacts its sympatric host, it triggers coral bleaching (i.e., substantial or partial loss of endosymbiotic dinoflagellate microalgae - commonly referred to as zooxanthellae - from coral tissues, and/or the loss or reduction of photosynthetic pigment concentrations within zooxanthellae) when the temperature reaches 29.5°C [22]. YB1 caused no pathogenicity in an allopatric coral from the Gulf of Eilat (Red Sea) at temperatures less than 24°C, triggered bleaching between 24 and 25°C, and caused tissue lysis at temperatures exceeding 25°C [35]. In another allopatric interaction involving a *P. damicornis* isolate from Lombok (Indonesia), no signs of infection were evident at temperatures up to 25°C, but at 28°C the bacteria penetrated the host tissues inducing rapid tissue lysis but no bleaching [33]. These results suggest that this bacterium has a high epizootic potential because of its ability to infect allopatric host populations and the various factors modulating its pathogenesis [32].

In this context, we used the infection model described above to investigate the relationship between temperature and bacteria (non-virulent and virulent), and their effects on coral physiology and pathogenesis. To this end, coral nubbins were exposed to stable or increasing temperature with or without bacterial addition, and the physiological changes caused at key stages of the interaction by the various treatments were assessed at the genome-wide scale using RNA sequencing (RNA-seq) approaches. We also used the quantitative reverse transcriptase polymerase chain reaction (q-RT-PCR) to study the response of 44 candidate immune genes during the stress response.

## Methods

### Biological material

The *P. damicornis* (Linnaeus, 1758) isolate used in this study was obtained from Lombok, Indonesia (Indonesian CITES Management Authority, CITES number 06832/VI/SATS/LN/2001-E; France Direction de l’Environnement, CITES number 06832/VI/SATS/LN/2001-I) and has been maintained in aquaria since the year 2001. Analysis of the first 600 bp of the mitochondrial ORF marker [36] showed that morphologically and genetically this *P. damicornis* isolate corresponds to the recently re-characterized *P. damicornis* clade [20], [37], [38]. For use in the experiments, coral explants (7 cm high, 6 cm diameter) were detached from the parent colony, and left to recover for a period of 1 month prior to use.

The coral pathogen *Vibrio coralliilyticus* strain YB1 [39]; CIP 107925, Institut Pasteur, Paris, France) was used to challenge or infect *P. damicornis* [22]. *V. coralliilyticus* was cultured as previously described [33].

### Stress protocol

As the objective of the study was to detect stress effects and to avoid the potential influence of inter-individual variability and genetic background on these effects, the experiments were performed using clones of the same *P. damicornis* isolate and a unique bacterial strain (YB1). Coral nubbins were randomly placed in 120 L tanks (n = 27 per tank) and acclimatized at 25°C for a period of two weeks prior to initiating the treatments. To distinguish the effects of bacterial stress (exposure to *V. coralliilyticus* under non-virulence conditions), thermal stress and bacterial infection (*V. coralliilyticus* virulence activated by temperature increase) on the coral host, three independent treatments and a control were established as previously described [33]. For the non-virulent treatment, *V. coralliilyticus* was regularly added to coral nubbins held at 25°C, which is a sub-virulent temperature in this host/pathogen interaction. For the virulent treatment, *V. coralliilyticus* was regularly added to coral nubbins while the temperature was gradually increased from 25°C to 32.5°C, triggering activation of bacterial virulence and infection. For the control, the coral nubbins were held at 25°C without added bacteria. For the thermal stress treatment, the coral nubbins were subjected to a gradual increase in temperature without the addition of bacteria, triggering thermal stress that induces bleaching. For all treatments and the control, three nubbins were randomly sampled every three days and immediately frozen and stored in liquid nitrogen. Coral health and the stability/breakdown of the coral symbiosis were monitored over time in each experimental group by assessing the density of zooxanthellae and visual monitoring, as previously described [33].

### cDNA library construction and high-throughput sequencing

Four cDNA libraries, corresponding to the control and treatment groups, were sequenced using Illumina GAIIx technology. One lane per library was used to generate an RNA-seq dataset of four 80-nucleotide paired-end short sequence reads. The samples for this analysis were collected at day 12, which was the last sampling occasion prior to the appearance of symptoms of bleaching or bacterial infection in the thermal stress and virulent treatments, respectively.

The total RNA from the 3 coral nubbins of each of the control and treatment groups was extracted using TRIzol reagent (Invitrogen), as previously described [40]. The cDNA libraries were then constructed following an established protocol [41]. Briefly, to obtain high quality mRNA, two cycles of oligo (dT) purification were performed using the Ambion Poly(A) Purification kit (Ambion). For the second round of purification, the mRNA purified in the first round was used in excess (4 µg mRNA per library). Subsequently, first strand cDNA synthesis was performed by combining 1 µg of the purified mRNA, 300 µg of random hexamer primers and the superscript III reverse transcription kit (Invitrogen) reagent. The excess of random primers enabled generation of first strand cDNA with an average length of 400 bp, and maximized coverage at the 5′ and 3′ ends of the mRNA. The second strand synthesis was performed using a combination of RNase H enzyme and DNA polymerase I (New England Biolabs). The resulting cDNA was purified using the QIAquick PCR purification kit (Qiagen). The amount and quality of nucleic acid obtained using this protocol was determined using a bioanalyzer and a nanodrop apparatus. The purified cDNA (1 µg) was used to generate each of the paired-end Illumina sequencing libraries. The libraries were prepared using Illumina adapter and PCR primers, according to previously published protocols [42], [43]. Libraries with an average insert size of 400–500 bp were isolated, and the concentration was adjusted to 10 nM. The samples (7 pM per sample) were loaded into separate channels of an Illumina GAIIx sequencer. The sequencing was performed at the Oregon State University Center for Genome Research and Biocomputing. The raw data (untreated reads) for all treatments are publicly available (http://www.ncbi.nlm.nih.gov/sra study accession number SRP029998).

### Differential gene expression analysis

To assess changes in gene expression induced by the various treatments, reads from each library were mapped against a previously assembled reference transcriptome [44]. This transcriptome was assembled *de novo* from 80-nucleotide paired-end short sequence reads based on 6 lanes of Illumina sequencing, and contained 72,890 contigs. Among these contigs, 27.7% and 69.8% were predicted to belong to the symbiont and the host transcriptomes, respectively. Each sequencing lane contained cDNA prepared from coral nubbins of the same genotype. The sequencing lanes were loaded with cDNA from corals exposed to: 1) thermal stress (lane 1); 2) *V. coralliilyticus* in a non-virulent state (lane 2); 3) *V. coralliilyticus* in a virulent state (lane 3); 4) a constant temperature of 25°C (lane 4); 5) a pH of 7.4 for 3 weeks (lane 5); and 6) a pH of 8.1 for 3 weeks. This reference transcriptome is accessible at http://2ei.univ-perp.fr/telechargement/transcriptomes/blast2go_fasta_Pdamv2.zip.

The mapping was conducted using Burrows-Wheeler Aligner (BWA), using the default options: aln -n = 0.04; aln -o = 1; aln -e = -1; aln -d = 16; aln -i = 5; aln -l = -1; aln -k = 2; aln -M = 3; aln -O = 11; aln -E = 4 [45]. As RNA-seq data are a function of both the molar concentration and the transcript length, the results of the mapping step were corrected and expressed as reads per kilobase per million mapped reads (RPKM; [46]). To identify significantly different gene expressions among the control and treatment groups, the MARS method (MA plot-based methods using a random sampling model) of the R package in DEGseq was used [47].

### q-RT-PCR

Quantitative real-time PCR (q-RT-PCR) was used to validate the expression profiles obtained from the MARS DEGseq analysis of the RNA-seq data. It was also used to measure expression levels of selected candidate genes during the non-virulent, thermal stress and virulent treatments. Total RNA was extracted and treated with DNase, and the poly(A) RNA was purified as described above. Approximately 50 ng of purified poly(A) RNA was reverse transcribed with hexamer random primers using ReverTAid H Minus Reverse Transcriptase (Fermentas). The q-RT-PCR experiments were performed using cDNA obtained from three coral nubbins per control and treatment group, as described previously [33]. For each candidate gene the level of transcription was normalized using the mean geometric transcription rate of three reference sequences encoding ribosomal protein genes from *P. damicornis* (60S ribosomal protein L22, GenBank accession number HO112261; 60S ribosomal protein L40A, accession number HO112283; and 60S acidic ribosomal phosphoprotein P0, accession number HO112666). The stable expression status of these three genes during biotic and abiotic stress has been demonstrated previously [33].

### Statistical analysis

Differences in gene expression data obtained using the RNA-seq analyses were analyzed for statistical significance using the MARS method, as described above [47]. Differences in transcript levels among experimental groups were considered statistically significant at *p*<0.0001. The statistical analyses used to identify biological processes (Gene Ontology term) that were significantly up- or down-regulated [48] were performed using Blast2GO software (version 2.6.4). Increases in processes were considered statistically significant at *p*<0.05. The hierarchical clustering of q-RT-PCR data was performed using Multiple Array Viewer software (version 4.8.1), with average linkage clustering using the Pearson correlation as a default distance metric. The normality of the RPKM distribution was assessed using the Kolmogorov-Smirnov test. As the data were not normally distributed, we used non-parametric statistical procedures. The Mann-Whitney U-test was used to compare the expression level between the core set of up-regulated genes and the core set of down-regulated genes. These statistical analyses were conducted using SPSS 10.0 (Kolmogorov-Smirnov and Mann-Whitney), and were considered statistically significant at *p*<0.05.

## Results

### Gene expression analysis and validation

**Figure 1:**
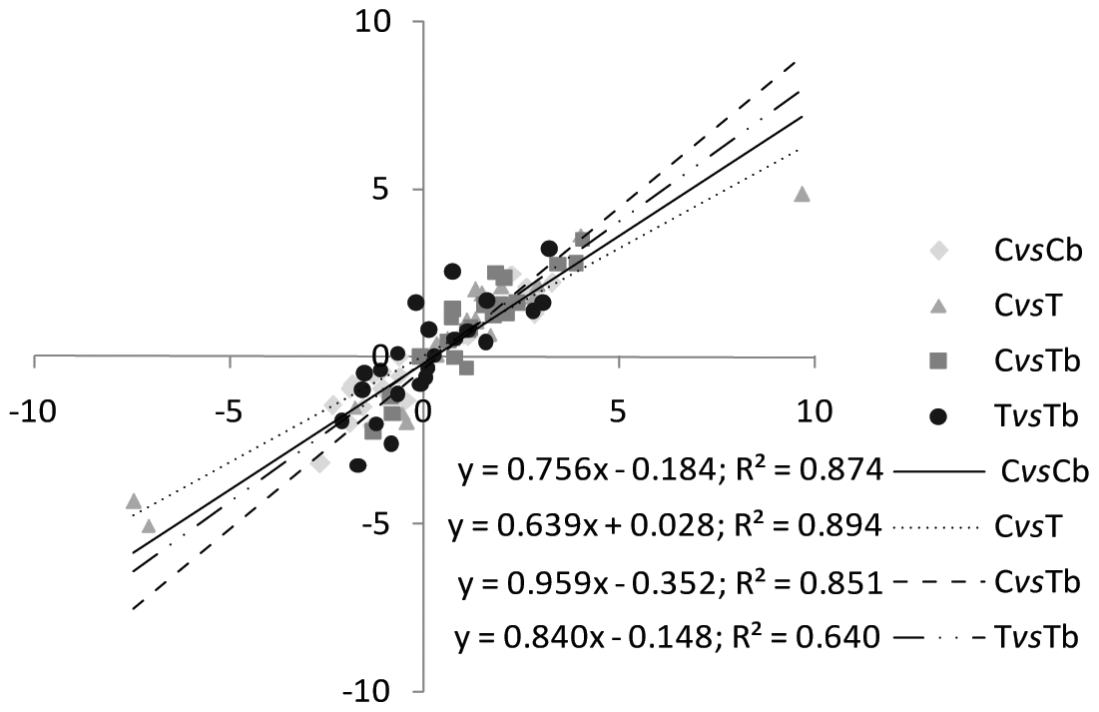
Validation of the RNA-seq approach using q-RT-PCR. Twenty-two genes were arbitrary selected, from highly up-regulated to highly down-regulated contigs. Their levels of expression were quantified by q-RT-PCR, and the results were compared with those obtained using the RNA-seq approach. The log2 changes in expression based on q-RT-PCR and RNA-seq analyses were closely correlated for all treatments, indicating the accuracy of the RNA-seq approach for quantification.

The global transcriptomic approach was conducted using RNA-seq methodology that was applied to samples collected 12 days following initiation of the various treatments. To distinguish the bacterial virulence effect from the thermal stress effect in the virulent treatment, in which corals were submitted to a combination of added bacteria and increased temperature, we used the same methods to compare the transcript content in the thermal stress and the virulent treatments. This comparison was called the virulence effect. For each treatments and comparison the results of the: i) Illumina sequencing, ii) quality control and filtering, iii) mapping to the reference transcriptome, and the iv) statistical approach (DEGseq) are summarized in the Table 1. To validate results from the RNA-seq analysis we used an alternative method (q-RT-PCR) for transcript quantification. For this analysis, 22 host transcripts were selected along the gradient of expression from highly up-regulated to highly down-regulated genes, based on RNA-seq data. A significant correlation between the log2-fold change in expression of the RNA-seq and the qRT-PCR data was obtained (log2-fold change qRT-PCR *vs.* log2-fold change RNA-seq: control *vs.* non-virulent, *r* ^2^ = 0.87 and *p*<0.0001; control *vs.* thermal stress, *r* ^2^ = 0.89 and *p*<0.0001; control *vs.* virulent, *r* ^2^ = 0.875 and *p*<0.0001; thermal stress *vs.* virulent, *r* ^2^ = 0.64 and *p*<0.0001; Fig. 1) and validated the results obtained through the RNA-seq analytical pipeline.

**Table 1:** Sequencing, filtering and gene expression analysis.

	Control	Non-virulent	Thermalstress	Virulent	Virulence (thermal stress vsvirulent treatment)
Total reads (millions)	27.7	20.4	17.0	19.3	
Reads passing quality filter (millions)	7.0	6.8	8.7	9.4	
Predicted host transcriptome mapped reads		88.4%	85.2%	87.3%	81.6%
Predicted symbiont transcriptome mapped reads		79.9%	78.0%	79.5%	75.8%
Significantly up-regulated genes		5,810	8,179	2.696	4.702
Significantly down-regulated genes		3.543	13.342	14.166	11,299
Predicted host genes significantly up-regulated		4713	3126	1578	4089
Predicted symbiont genes significantly up-regulated		1097	5053	1118	613
Predicted host genes significantly down-regulated		2401	11735	11382	5272
Predicted symbiont genes significantly down-regulated		1142	1607	2784	6027

### Specific biological processes modulated in the various experimental conditions

A Gene Ontology (GO) term enrichment analysis [48] was performed on the sets of transcripts showing a significant change in expression. The results showed that in response to the non-virulent treatment, 12 biological processes were significantly enriched in the set of up-regulated genes (*p<*0.05; Table 2), and 11 in the set of down-regulated genes (*p<*0.05; Table 3). In response to the thermal stress treatment 20 biological processes were significantly more represented in the set of up-regulated transcripts relative to the control (*p<*0.05; Table 2) and 20 from the set of down-regulated genes (*p<*0.05; Table 3). In response to the virulent treatment six biological processes were significantly enriched (*p<*0.05) from the set of up-regulated genes (*p<*0.05; Table 2) and 20 from the set of down-regulated genes (*p<*0.05; Table 3).

**Table 2:** Biological functions significantly (*p*<0.05) enriched in the sets of up-regulated genes.

Treatments	Non-virulent		Thermal stress		Virulent		Virulence	
GO Term	Up-regulated	Not-regulated	Up-regulated	Not-regulated	Up-regulated	Not-regulated	Up-regulated	Not-regulated
alcohol metabolic process			20	91				
aromatic compound biosynthetic process			6	9				
ATP synthesis coupled proton transport	10	43						
bioluminescence	6	10						
biosynthetic process							64	899
cellular amino acid biosynthetic process			18	60				
cellular aromatic compound metabolic process	10	46						
cellular component biogenesis			27	109				
Cellular metabolic process							190	2523
Cellular protein modification process	81	899						
cofactor metabolic process			24	58				
fatty acid metabolic process			7	15				
gene expression							57	481
generation of precursor metabolites and energy			88	95	68	113	45	187
heterocycle metabolic process			59	261				
innate immune response	10	45						
ion homeostasis	7	23						
ion transport			52	249				
macromolecular complex subunit organization			25	86				
metabolic process			726	3466	253	4022		
microtubule-based process			25	117				
nitrogen compound metabolic process			127	833				
nucleoside phosphate metabolic process			48	231				
nucleotide biosynthetic process	19	119						
organic acid metabolic process			43	202				
phosphorus metabolic process	68	871						
phosphorylation	57	411						
photosynthesis			67	47	65	54	39	74
protein stabilization			3	0				
proton transport	14	80						
regulation of gene expression			5	12				
response to chemical stimulus					7	39		
response to oxidative stress			10	24	7	29		
RNA processing	14	69						
small molecule metabolic process			119	499				
translation							45	352
transmembrane transport	36	271			19	284		

**Table 3:** Biological functions significantly (*p*<0.05) enriched in the sets of down-regulated genes.

Treatments	Non-virulent		Thermal stress		Virulent		Virulence	
GO Term	Down-regulated	Not-regulated	Down-regulated	Not-regulated	Down-regulated	Not-regulated	Down-regulated	Not-regulated
apoptotic process			15	33				
biological regulation			162	646				
biosynthetic process	66	881			232	702		
cell surface receptor signaling pathway					38	114		
cellular biosynthetic process			186	662				
cellular component assembly at cellular level					27	74	33	79
cellular component organization or biogenesis			52	186	61	185		
cellular macromolecule biosynthetic process					165	394		
cellular macromolecule metabolic process							337	1456
cellular respiration			16	42				
cellular response to stress	7	68						
chromosome organization			18	29	21	26		
cofactor biosynthetic process			13	33				
cofactor metabolic process							25	59
gene expression	59	476	154	370	174	357		
generation of precursor metabolites and energy	56	128						
glutamine metabolic process					5	6		
hydrogen transport							31	63
immune response			19	49	20	47		
macromolecule metabolic process			407	1758				
metabolic process	220	4120						
microtubule-based process							40	115
neurotransmitter transport			11	9	10	13		
nitrogen compound metabolic process							60	178
nucleic acid metabolic process			77	306				
nucleobase-containing compound catabolic process					32	91		
organic acid transport					8	12		
peptidyl-amino acid modification			8	15				
phospholipid metabolic process			9	20				
phosphorylation			95	400				
photosynthesis	37	38						
primary metabolic process			524	2501	659	2422		
protein folding					47	108	46	113
protein metabolic process	93	1790	337	1477	422	1421		
protein polymerization							30	36
protein targeting	7	8			7	8		
regulation of cell death					12	22		
regulation of metabolic process							25	65
regulation of phosphate metabolic process					12	21		
regulation of protein phosphorylation					11	21		
response to DNA damage stimulus	7	68						
response to stimulus			153	640				
S-adenosylmethionine metabolic process	2	9						
signaling			113	442				
translation	55	339	119	270	137	257		

We then investigated whether there were conserved up- or down-regulated genes in response to all treatments. This analysis showed that 229 genes were significantly up-regulated in response to the non-virulent, thermal stress and virulent treatments, while 1372 were significantly down-regulated in all experimental groups. All the genes showing conserved regulation among groups are referred to as core response genes. Four biological processes were significantly enriched in the set of core up-regulated genes (*p*<0.05; Table 4), and 15 from the core down-regulated genes (*p*<0.05; Table 4). To distinguish the bacterial virulence effect in the virulent treatment, in which corals were submitted to a combination of added bacteria and increased temperature, the same enrichment analysis was performed comparing transcriptomic data obtained from the thermal stress and the virulent treatments. This enabled identification of six enriched biological processes from the set of genes up-regulated in the virulence treatment (*p<*0.05; Table 4) and nine in the set of genes down-regulated (*p<*0.05; Table 4).

**Table 4:** Biological functions significantly (*p*<0.05) enriched in the sets of up-regulated and down-regulated core genes**.**

Regulation	Up-regulated		Down-regulated	
GO Term	Up-regulated	Not-regulated	Down-regulated	Not-regulated
transmembrane transport	7	299		
cellular biosynthetic process	7	865		
localization	7	873		
ATP biosynthetic process	5	78		
cellular process			66	4094
primary metabolic process			58	3079
protein metabolic process			38	1848
biosynthetic process			36	915
gene expression			32	503
macromolecule biosynthetic process			31	542
translation			30	364
nucleic acid metabolic process			11	417
protein localization			6	223
cellular respiration			4	54
developmental process			3	47
protein targeting			3	15
sulfur compound biosynthetic process			2	24
cellular modified amino acid metabolic process			2	29
nucleoside biosynthetic process			5	102

The annotation and expression levels of the genes belonging to each enriched GO category are shown in Table S1 & S2.

### Selection of immune candidate genes and expression analysis

As the enrichment analysis revealed a clear effect of the various treatments on the immune function, we investigated and compared the expression of putative *P. damicornis* immune genes, amongst the various treatments. These genes, annotated manually or using Blast2GO, corresponded to the immune toolbox of *P. damicornis*. They included genes encoding proteins involved in recognition (e.g. TLR and lectins), signaling pathways (e.g. NF-kB, AP1/ATF, c-Jun), complement system (e.g. C3, C-type lectin, and membrane attack complex), prophenoloxidase cascade (e.g. prophenoloxidase activating enzyme, laccase), leukotriene cascade (e.g. 5-lypoxigenase, leukotriene A4 hydrolase), antimicrobial molecules (e.g. LPBPI and antimicrobial peptides) and ROS scavengers (e.g. peroxidases, catalases, GFP-like molecules). The results supporting the annotation of these genes are presented in the Table S3.

**Figure 2:**
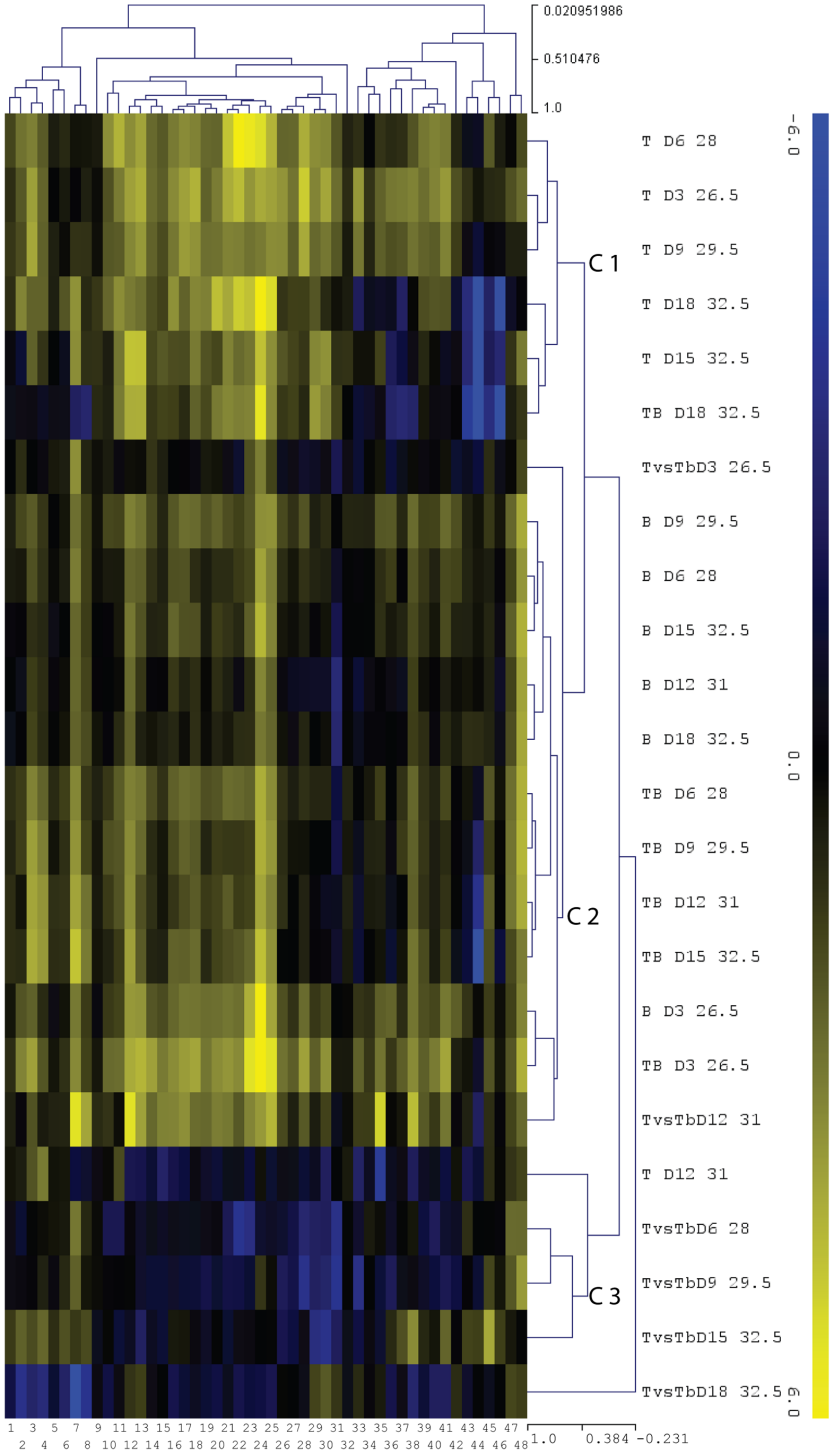
Expression of innate immune candidate genes. The data included q-RT-PCR results for nubbins sampled during the non-virulent, thermal stress, virulent and bacterial virulence (thermal stress *vs.* virulent treatment) treatments (days 3, 6, 9, 12, 15 and 18). Quantification was normalized to the control conditions for the non-virulent, thermal stress and virulent treatment, and with results for colonies sampled at the same temperature as that for the thermal stress *vs.* virulent comparison (bacterial virulence effect only). The results are presented as a log2-fold change in expression. The hierarchical clustering of the q-RT-PCR data was done using Multiple Array Viewer software (version 4.8.1), with average linkage clustering based on the Pearson correlation as a default distance metric. Cluster C1 represents the response to thermal stress, cluster C2 represents the response to bacteria (non-virulent and virulent), and cluster C3 represents the response to the virulence of the bacteria. The numbers at the bottom of the figure correspond to the following genes: 1, epsilon isoform 1 (housekeeping control); 2, MASP3; 3, peroxidase2; 4, peroxidase1; 5, cyclin d2 (housekeeping control); 6, preprotein translocase SecY subunit (housekeeping control); 7, laccase; 8, prophenol oxidase activating enzyme; 9, ubiquitin-conjugating enzyme E2 (housekeeping control); 10, 5-lypoxigenase; 11, catalase1; 12, nucleoredoxin; 13, TAK1; 14, MKK7; 15, MKK4; 16, JNK; 17, TRAF6; 18, IKBa; 19, NF-kB; 20, TIR2; 21, AP1; 22, ATF; 23, Bf; 24, C3; 25, MASP1; 26, catalase2; 27, MKK3/6; 28, IKK; 29, p38; 30, LRR2; 31, apextrin; 32, SOD1; 33, leukotriene A4 hydrolase; 34,Leukotriene C4 synthase; 35, MyD88; 36, GFP-Like2; 37, TIR3; 38, SOD2; 39, MEKK1; 40, LPBPI; 41, LRR-TIR-IGG; 42, GFP-Like1; 43, Tx60A2; 44, Tx60A1; 45, PdC-Lectin; 46,damicornin; 47, phospholipase A2; 48, mytimacin-like.

To assess the impact of each treatment on the regulation of these genes, their expression was assessed every 3 days during the experimental period, using q-RT-PCR. To confirm that the observed regulation of gene expression was not because of physiological collapse of the coral or an experimental artifact, four housekeeping genes were also monitored during the experimental period. These genes were selected from amongst host genes that were not regulated in any treatment. As expected, the results showed that their expression remained stable in all treatments (Fig. 2; Table S4).

**Figure 3:**
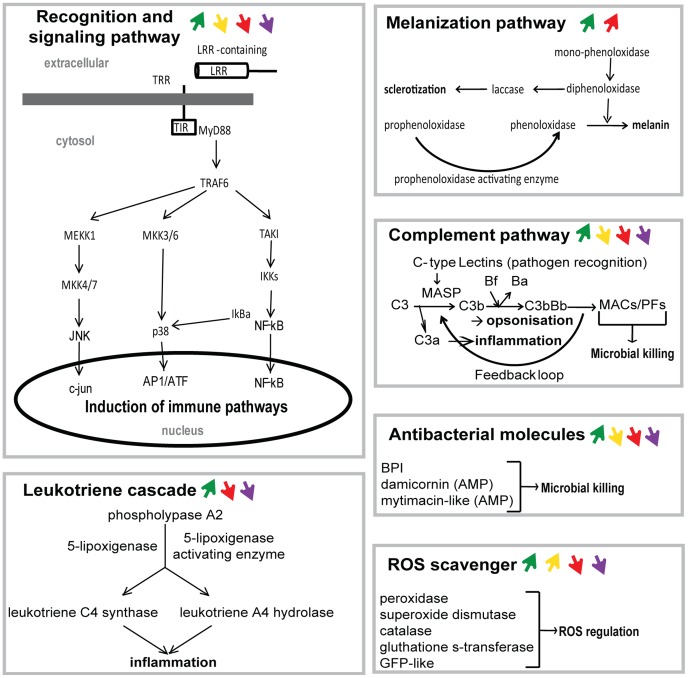
Schematic representation of the innate immune pathways monitored by q-RT-PCR, and their main response to each treatment. Reconstitution of the immune pathways identified in previous studies and from the present study (see Table 5 for references). Arrows highlight the average response (if any) of each pathway to each treatment or comparison. Green arrow: response to the non-virulent treatment; yellow arrow: response to the thermal stress treatment; red arrow: response to the virulent treatment; violet arrow: response to the virulence effect (the comparison between the thermal stress and the virulent treatment).

A hierarchical clustering approach was used to create sample and gene trees for the 24 samples (six for each treatment) and the 48 genes (44 immune genes and four housekeeping genes; Fig. 2). Three distinct clusters were identified. The first cluster mainly represented the response to thermal stress (cluster C1; Fig. 2), and contained two subgroups of genes that were highly modulated. The first subgroup comprised genes displaying a high level of down-regulation, including those encoding the antimicrobial peptide damicornin (average 40.0-fold decrease; Fig. 2), the mannose binding lectin PdC-Lectin (average 6.9-fold decrease; Figs 2 and 3), and two members of the membrane attack complex (Tx60A1 and A2; average 91.2-fold decrease; Fig. 2). The second subgroup comprised genes displaying a high level of up-regulation, including those encoding a putative MASP1, putative C3 and Bf components of the complement pathway (average 26.4-fold increase; Figs 2 and 3), and two immune-related transcription factors ATF and AP1 (average 19.3-fold increase; Figs 2 and 3). The second cluster (cluster C2; Figs 2 and 3) corresponded to genes involved in the response to bacteria (non-virulent and virulent treatments). It included genes subject to down-regulation (e.g. a gene encoding a putative apextrin protein; average decrease 3.2-fold; Figs 2 and 3), and some showing up-regulation (e.g. genes encoding prophenoloxidase activating enzyme and laccase, which are two key elements of the prophenoloxidase pathway; average 8.1-fold increase; Figs 2 and 3). The third cluster (cluster C3; Fig. 2) corresponded to genes involved in the response to the virulence of the bacteria. This cluster was mainly characterized by general down-regulation of the innate immune recognition and signaling pathways; the genes encoding the LRR and TIR domains containing proteins, TAK1, IKK, NF-kB, MKK3/6, P38, IkBa, AP1/ATF and MKK4/7 were all down-regulated by an average factor of 4.1 (Figs 2 and 3).

In relation to specific biological functions, the q-RT-PCR experiments provided noteworthy results for several immune genes and the main ones are highlighted below. For antimicrobial effectors, we found that expression of the damicornin, mytimacin-like and LBP–BPI genes was decreased in the thermal stress and virulent treatments (Figs 2 and 3). The two genes of the prophenoloxidase pathway (prophenoloxidase activating enzyme and laccase) were co-up-regulated in the presence of bacteria (non-virulent and virulent treatments) whereas in response to bacterial virulence, there were strongly down-regulated at day 18. The complement pathway (PdC-lectin, MASP1 and 2, C3 and Bf) was mainly up-regulated by the presence of non-virulent bacteria and during the virulent treatment. Members of the membrane attack complex Tx60 A1 and A2, and apextrin (a downstream component of the complement pathway; Fig. 3) were strongly down-regulated under temperature stress conditions (thermal stress and virulent treatment). The expression of some key components of the backbone of the recognition and signaling pathways (LRR, TIR, Myd88 and TRAF6) was disturbed during the virulent, virulence and thermal stress treatments. The downstream pathways of this backbone (NF-kB, ATF/AP1 and JNK) showed a similar trend of regulation in response to the non-virulent bacteria, thermal stress and virulent bacteria. In response to bacterial virulence the NF-kB and ATF/AP1 pathways were completely down-regulated (Figs 2 and 3). The leukotriene cascade responded mainly to temperature stress by general up-regulation of its component. Among the genes encoding ROS scavengers, two groups could be clearly distinguished. The first group comprised ‘classical’ ROS scavengers (peroxidase, catalase, superoxide dismutase and nucleoredoxin), which showed strong up-regulation in response to thermal stress, non-virulent bacteria and virulent bacteria. The second group (encoding GFP-like protein); was initially up-regulated by thermal stress but then down-regulated at higher temperature (Figs 2 and 3). In response to the non-virulent bacterium these two genes were up-regulated, but were down-regulated immediately following initiation of virulence in the bacterium (virulent treatment, days 6–18; Figs 2 and 3).

## Discussion

By combining the analysis of natural coral/*Vibrio* interaction, experimental exposures of corals to the bacteria, global transcriptomic studies (RNA-seq) confirmed by q-RT-PCR, we showed that thermal stress induces a general decrease in coral gene expression; this included decreased expression of immune genes, hence reducing the immune capacities of the coral. We also found that virulent bacteria triggered a marked suppression of host immunity. The RNA-seq approach unveiled a large range of transcripts expressed either by the coral host or by its dinoflagellate symbionts (entire holobiont response). A clear illustration of the response of the symbiont was the down-regulation of photosynthesis genes and for the host, the up-regulation of immune genes in response to the non-virulent treatment). In general it was possible to assign transcripts to either the host or the symbiont partner (as explained in the methods section), although for some genes the discrimination was not always obvious. Assembled genomes for the two partners in the symbiosis are lacking, and dinoflagellate genomes are known to contain numerous copies of orthologous genes, which can be divergent. The latter factor increases the complexity of the transcriptome [49]–[52] and can make assignment difficult. In addition, our transcriptome probably contained xeno-contaminant sequences from bacteria, eukaryotic species and RNA viruses [53], which are part of the coral holobiont [54].

### The sophisticated ancestral immune toolkit responds to experimental bacterial exposure

The immunity of scleractinian corals is an expanding field in basic research because of the basal position of cnidarians in the eumetazoan tree of life, and its relevance to understanding the evolution of defense systems against pathogens. It is also crucial to understand the immune capabilities of corals, because of the increase of coral epizootics. In this context, most past studies have focused on the identification of immune pathways and effectors, mainly through searches for orthologous genes in the expanding genome and transcriptome databases. These studies have revealed that the common ancestor of animal species contained several immune genes and pathways that are also present in higher invertebrate and vertebrate species (Table 5). This discovery led to the hypothesis that early eumetazoan species had an ancestral immune toolkit [61], [81], which evolved to the more sophisticated systems found in higher lineage. This hypothesis remains to be proven because the functions of the putative immune genes and their involvement in immunity remain to be investigated [82]. Demonstration for this hypothesis was not trivial in our non-model organisms. Indeed, several immune genes are involved in a number of cellular processes (e.g. NF-kB transcription factor), but demonstrating their involvement in immunity will require knock-out or knock-down approaches that will have to be developed for non-model organisms, including corals. In this context, a targeted and organized response to a pathogen could be considered initial evidence that these genes and pathways are directly involved in the immune response. Some of the genes and proteins have been shown to respond to experimental infection or to pathogen elicitors (Table 5), but studies at the entire transcriptome level have not occurred. The present study partly addressed this shortcoming by showing that most of the known immune factors in the coral were modulated following a natural bacterial challenge. Indeed, we observed co-regulation of genes belonging to the same pathways, including genes for: (i) MASP1, C3 and Bf in the complement pathway; ii) the prophenoloxidase activating enzyme and laccase in the melanization pathway; and (iii) proteins involved in signaling pathways, including the transcription factors NF-kB and JNK (Fig. 3). In summary, the results of this study, and recent studies of *A. millepora* [83] and *Gorgonia ventalina* [84] indicate that corals are able to mount an immune response using an ancestral immune toolkit.

**Table 5:** Summary of the cnidarian immune genes identified.

Immune function	Gene/protein	References
Recognition	Lectins, integrins, Toll-like receptors	[33], [40], [55]–[65]
Signaling	NF- k B, AP1/ATF, JNK, Myd88, MAPKs	[64], [66], [67]
Complement	C3, mannose binding lectins, MASPs	[40], [60]–[62], [68]–[70]
Melanization	Laccase, phenoloxidase, prophenoloxidase	[71]–[78]
Antimicrobial activity	Hydramacin-1, Periculin1, Aurelin, Damicornin, LBP-BPI, mytimacin-like	[34], [55], [79], [80] this study
Leukotriene cascade	Phospholipase A2, 5-lipoxigenase, leukotriene C4-synthase, leukotriene A4-hydrolase	This study

### The effect of non-virulent bacteria

In the presence of the non-virulent bacteria, we found that innate immune pathways (Table 2 and Fig. 2) were activated in the coral, which is consistent with previous data showing that non-virulent *V. coralliilyticus* triggers an immune response [33], [34]. The absence of bacteria in host tissues during the non-virulent treatment [33] suggests that the coral either: (i) detected the presence of bacteria, and the coral cells developed an immune response directed against non-internalized bacteria; or (ii) was able to develop an efficient immune response that killed all bacteria on entry.

The mechanisms underlying the recognition of *V. coralliilyticus* by *P. damicornis* remain to be identified. However, in *Hydra* spp. (also a cnidarian) the unconventional pathogen sensors HyLRR, which are expressed at the surface of epithelial cells, interact with an intracellular HyTRR (a TIR domain-containing protein) during immune challenge [55], and this leads to the expression of an AMP (periculin-1). These LRR and TRR molecules were identified in the *P. damicornis* transcriptome, suggesting that *P. damicornis* can detect *V. coralliilyticus* using similar mechanisms. The ability to mount an immune response has been reported in numerous cnidarian species exposed to lipopolysaccharide (LPS) including *Montastraea faveolata*, *Stephanocoenia intersepta, Porites astreoides* and *Acropora millepora* [70], [77]. However, our coral/pathogen model is the first to have enabled an association to be made between the absence of infection and an organized up-regulation of multiple and interlinked pathways that reflect the three key steps in the immune response: i) pathogen recognition; ii) signal transduction; and iii) effector responses (Figs 2 and 3).

### The thermal stress effect

Consistent with several previous studies (for review, see; [85], we found that the response to thermal stress is characterized by up-regulation of several heat shock proteins (HSPs) and genes encoding ROS scavengers. However, we showed that this was accompanied by significant down-regulation of genes involved in innate immunity and apoptosis (Table 3). These observations support the hypothesis that high temperature favors infection because it impacts coral immune function [86]–[90]. However, this phenomenon may not occur with all immune genes. Indeed, our candidate gene approach showed that several immune genes were up-regulated in the thermal stress treatment (Fig. 2). Similar results have been reported in previous studies investigating the immune abilities of corals in response to thermal stress. Prophenoloxidase activity was shown to be higher in thermally stressed *M. faveolata* corals relative to healthy or diseased corals, whereas the opposite occurred for antibacterial and lysozyme-like activities [71]. Such results raise questions about the uniform nature of thermal stress effects on the coral immune response, and challenge the hypothesis that high temperature negatively impacts all coral immune functions and always favors infection [86]–[90]. Nevertheless, we showed that several encoding pattern recognition receptors (PRR; Toll-like, LBP–BP and mannose-binding lectin) followed a general trend of down-regulation, as did key factors in the downstream signaling pathway, including Myd88 (Figs 2 and 3). Other essential components of the immune response, and more particularly some effectors, were also down-regulated (damicornin, TX60A-1 and TX60A-2) during thermal stress, which may significantly alter the immune capabilities of the coral. Thus, the combination of our results and those reported previously suggest that, even if the immune abilities of *P. damicornis* are not completely inhibited by thermal stress, the changes induced by increased temperature reduce the capacity of the coral to mount an efficient immune response, thus facilitating pathogen colonization of host tissues.

### The virulence +/− thermal stress effect

Analysis of the transcriptomic changes between the virulent treatment and the control (thermal stress+virulence effect) or the thermal stress (virulence effect) treatment indicated profound remodeling of the transcriptome. Coral immune function was highly affected by the infection process, and many genes were down-regulated, particularly following internalization of the bacteria. This finding is consistent with evidence from a previous study of *P. damicornis*, which showed down-regulation of the gene encoding the AMP damicornin during infection by *V. coralliilyticus* [34]. Nevertheless, this down-regulation response is not absolute, as revealed using the candidate gene approach, which demonstrated the up-regulation of some genes. This approach also highlights that the coral response to the virulent and non-virulent bacteria was very similar (Fig. 2; cluster C2). This suggests that the response is mediated following interaction with the bacteria, and is sufficient to neutralize the bacteria under non-virulent conditions, but not under virulent conditions. This shows that under virulent conditions the bacteria were able to circumvent the coral response and to establish the pathogenic intracellular and intra-vesicular form, which involves localization that facilitates protection of the bacteria from systemic immune attack [26], [33]. This strategy is also used by the oyster (*Crassostrea gigas*) pathogen *V. splendidus*: following internalization in immune cells this bacterium manipulates and evades host defenses by preventing acidic vacuole formation and limiting ROS production [91]. At high temperature and at the same stage of infection, *V. coralliilyticus* has also been shown to be able to express a series of putative virulence factors involved in host degradation, secretion, antimicrobial resistance and transcriptional regulation, which may help this pathogen to survive and spread in the intracellular environment [31], [32].

### The greater the stress, the greater the transcriptomic disturbance: a trade-off mechanism?

Based on the symptoms evident in each treatment, exposure to the bacteria under high temperature conditions triggering virulence resulted in greater stress to the coral than exposure to thermal stress alone, which in turn was more stressful than exposure to the non-virulent bacteria. In the virulent treatment tissue lysis and death of the coral colonies occurred within 18 days, in the high temperature treatment the symbiosis broke down and coral bleaching occurred within 18 days, and no symptoms were observed in the treatment involving non-virulent bacteria [33]. Our RNA-seq results showed that 12 days following initiation of the treatments there was a clear correlation between the intensity of the stress and the number of down-regulated genes: the greater the stress, the greater the down-regulation (Table 1). Extensive down-regulation in response to environmental stressors is often reported in transcriptomic studies, but most research has focused on biological functions that are expected to be affected, rather than using the global analysis of the physiological status of the impacted organism. Indeed, it is easier and more straightforward to focus on down-regulated genes clearly associated with specific functions than to interpret the modulation of numerous genes associated with diverse, general biological processes. Nonetheless, the phenomenon of widespread down-regulation during stress responses has been verified through genome-wide transcriptomic studies in corals and other organisms exposed to various environmental stressors [44], [92]–[97]. This suggests that down-regulation is a conserved phenomenon under stress conditions.

In this context, the coral stress response was recently compared with the environmental stress response (ESR) of a budding yeast [98], which involves a conserved, extreme, rapid, and genome-wide response to a broad set of environmental stressors that trigger a common response among a large set (approximately 900) of genes [99]. In corals the core stress response involves the up-regulation of HSP and ROS scavenger encoding genes, and disruption of the expression of genes involved in Ca^2+^ homeostasis, cytoskeleton organization, cell signaling and transcriptional regulation [98]. However, as was found in the yeast, in which approximately 66% of the genes of the ESR were down-regulated [99], the broad down-regulation observed in corals exposed to various stressors may also be a fundamental part of the coral stress response. This hypothesis is supported by parallels between the coral response in our study, and the main biological processes down-regulated in the yeast ESR. As in the yeast ESR [99], our GO enrichment analysis (Table 4) suggested a general down-regulation of what are usually considered to be housekeeping biological processes, including translation, nucleic acid metabolic processes, secretion, regulation of gene expression and others biosynthetic processes. These housekeeping functions are generally very costly, and monopolize most energy production and the cell’s transcriptional and translational machinery [100]. Under stress conditions, down-regulation may help to minimize energetic expenditure, enabling rapid and efficient responses to the new environmental conditions [101]. In yeast, this energetic economy was shown to affect functions that are not essential for survival, but also involved the selective down-regulation of genes encoding high molecular weight and/or highly expressed proteins [101]. Demonstrating such phenomena in corals is difficult because of the lack of tools enabling complete transcriptome analysis. However, the results obtained in our study for the host core genes were consistent with this hypothesis. Indeed, we found that under the control conditions the core coral genes were more represented among the down-regulated genes (RPKM average, 789.40 reads) than the up-regulated genes (RPKM average, 546.74 reads; Mann-Whitney U test, *p*<0.01).

## Conclusion

Taken together, our results show that scleractinian corals have an immune system that is able to respond to pathogenic agents, and support the “sophisticated ancestral immune toolkit” hypothesis [61], [81], [102]. The results also have important implications for understanding the immune system of ancestral metazoans, and its evolution and conservation through the eumetazoan lineage. Our results demonstrate that thermal stress alters immune capacities, especially during recognition and antibacterial processes, and that this probably facilitates pathogenesis. However, bacterial virulence and the intracellular localization of the pathogen seem to be the major factors responsible for pathogenesis, especially through an immuno-suppressive effect that may decrease the efficiency of the immune response, and lead to bacterial proliferation and physiological collapse of the coral. In addition to effects on immunity, thermal stress induces a strong and energetically costly response that may weaken the coral and favor infection by specific or opportunistic pathogens, through a trade-off mechanism. In summary, the absolute effect of thermal stress on the coral is less than that of the virulent bacteria during pathogenesis, but is a clear facilitator of infection.

## Supporting Information

Table S1 **Detailed results of the GO term enrichment analysis for the up-regulated set of genes.**
(XLS)

Table S2 **Detailed results of the GO term enrichment analysis for the up-regulated set of genes.**
(XLSX)

Table S3 **Candidate innate immune genes, Annotation, Contig name, Sequence, Primer, BlastX results and protein domain.**
(XLSX)

Table S4 **Expression of innate immune candidate genes, numerical values.**
(XLSX)
